# Correction: Epistasis in genomic and survival data of cancer patients

**DOI:** 10.1371/journal.pcbi.1006887

**Published:** 2019-02-27

**Authors:** Dariusz Matlak, Ewa Szczurek

There are errors in the Funding statement. The correct Funding statement is as follows: This work was supported by grants 2015/16/W/NZ2/00314 to DM and ES and 2015/19/P/NZ2/03780 to ES from the National Science Centre, Poland https://www.ncn.gov.pl/?language=en. This project has received funding from the European Union’s Horizon 2020 research and innovation programme under the Marie Sklodowska-Curie grant agreement No 665778. The funders had no role in study design, data collection and analysis, decision to publish, or preparation of the manuscript.
