# Supplementary figures and images for: Epistasis in genomic and survival data of cancer patients
Source: PLoS Comput Biol. 2017 Jul 5;13(7):e1005626. doi: 10.1371/journal.pcbi.1005626 (PMC5517071; doi:10.1371/journal.pcbi.1005626)

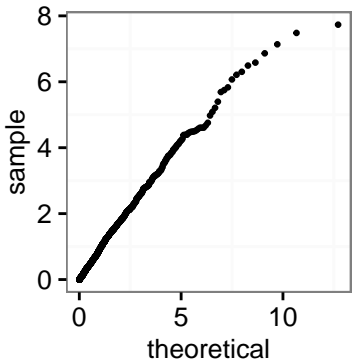

Supplement: S1 Fig — Theoretical quantiles of the χ12 distribution (x-axis) agree with the quantiles of the empirical distribution of −2log λ computed for data simulated from the null with 66% censored cases (y-axis). (PDF) [file pcbi.1005626.s001.pdf]

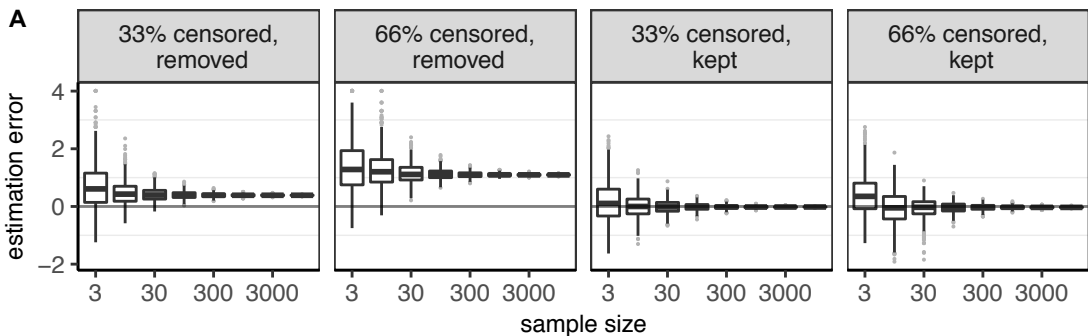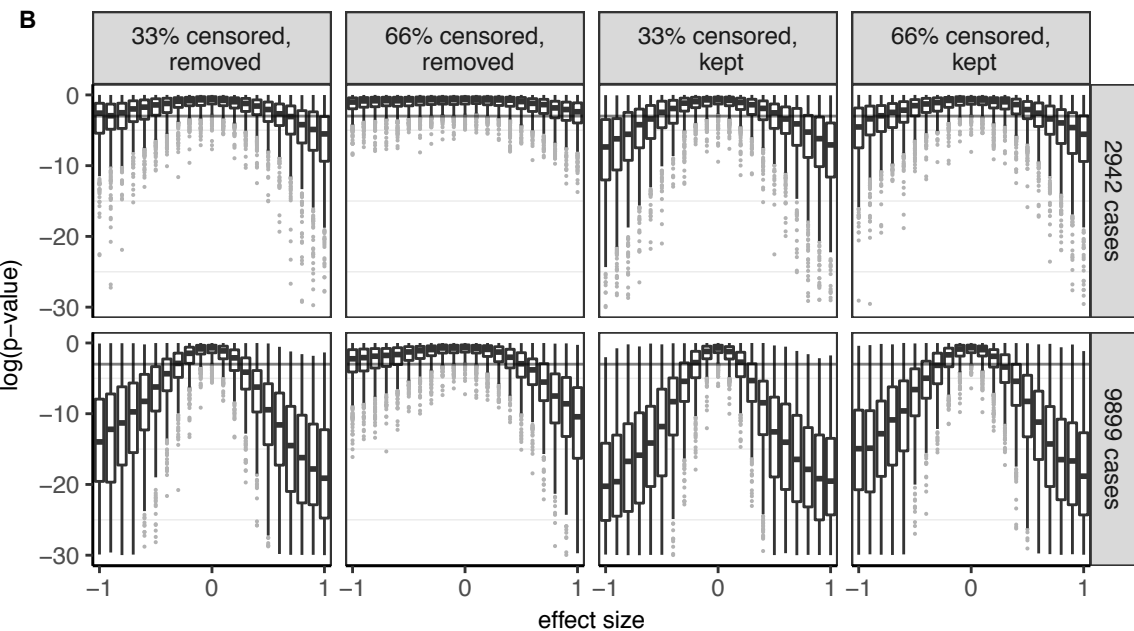

Supplement: S2 Fig — Figure panels are as in Fig 2, but here the data were simulated for G(t) estimated from survival times sampled from a truncated exponential distribution (up to 40 years survival, as observed in patient data). The results obtained with this alternative choice of the reference survival function are similar to those presented in Fig 2. A Box plots showing 25th, 50th and 75th percentiles (horizontal bars), and 1.5 interquartile ranges (vertical line ends) of log true to estimated fitness ratios (y-axis) as a function of sample sizes (x-axis), in the case when 33% and 66% of patients are censored and removed from the samples (first and second column, respectively) and when 33% and 66% are censored and kept (third and fourth column). Gray lines at 0 mark the level where the estimated equal the true parameter values. B Box plots of SurvLRT p-values as a function of epistatic effect size, for two different patient cohort sizes (rows). Gray lines mark the p-value 0.05. Columns as in A. (PDF) [file pcbi.1005626.s002.pdf]

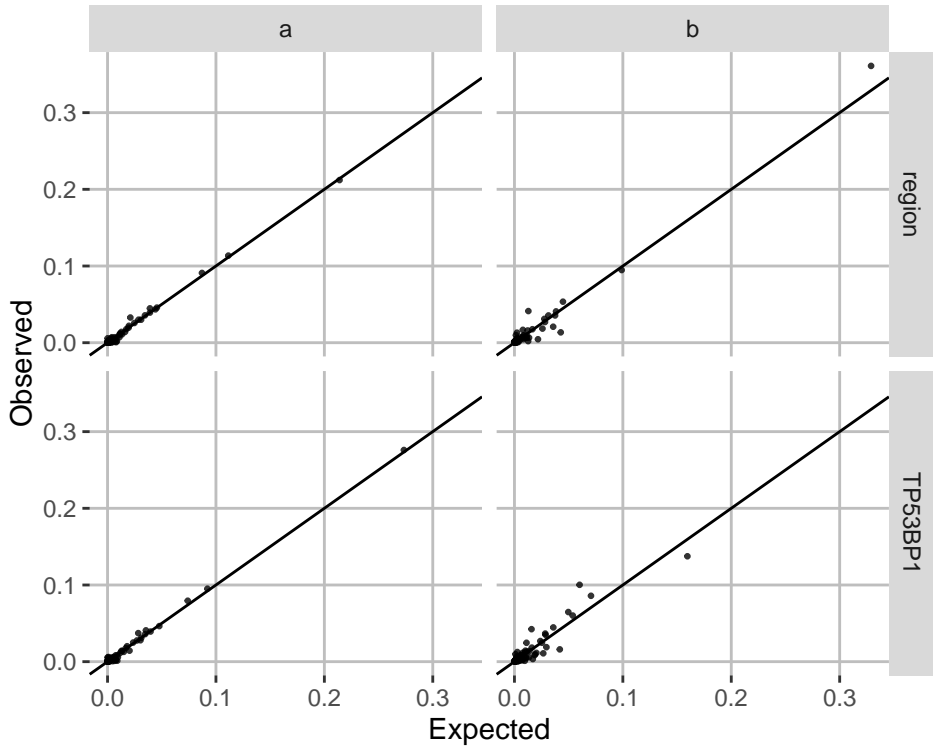

Supplement: S3 Fig — Each plot corresponds to one SurvLRT test. Points: proportions of the number of patients with each cancer type and carrying each of genotypes in the considered test to the total number of patients, as observed in the data (y-axis) versus the proportions expected given no cancer type bias, when the cancer types are distributed uniformly across the genotypes (x-axis). The line is where the points should arrange if the observed and expected proportions were equal. The colums correspond to the tests for triple epistasis type a and b, respectively, The two rows correspond to the tests where the triple epistasis was tested for the interaction between BRCA1, PARP1 (in both rows), conditional on the alteration status of either the newly identified biomarker region (top row) or the known biomarker TP53BP1 (bottom row). (PDF) [file pcbi.1005626.s003.pdf]
