# Supplementary material for: Epistasis in genomic and survival data of cancer patients
Source: PLoS Comput Biol. 2017 Jul 5;13(7):e1005626. doi: 10.1371/journal.pcbi.1005626 (PMC5517071; doi:10.1371/journal.pcbi.1005626)
Supplement: S1 Appendix — (PDF) [file pcbi.1005626.s009.pdf]

Epistasis in genomic and survival data of cancer patients

Dariusz Matlak[1], Ewa Szczurek[1,*]
[1] Faculty of Mathematics, Informatics and Mechanics, University of Warsaw, Warsaw, Poland,
[*] E-mail: szczurek@mimuw.edu.pl

Appendix

Formulae for the likelihood ratio and parameter estimators. Assume the settings from the *Methods* section in the main text. Recall that the random variable T denoting a healthy individual's lifetime has survival function $G(t)$, and assume it has a density f. With the model assumption that for patients with tumor genotype g the survival function is given by $G_g(t) = G(t)^{\Delta_g}$, the corresponding distribution function F_g reads

$$F_g = 1 - (1 - F(t))^{\Delta_g}.$$

Thus, the density is given by

$$f_g = \Delta_g (1 - F(t))^{\Delta_g - 1} f(t) = \Delta_g G(t)^{\Delta_g - 1} f(t).$$

Therefore, the likelihood of the survival data $L(\Delta; \bar{t}, \tilde{t})$, for the uncensored and censored cases, denoted $\bar{t}$ and $\tilde{t}$, respectively, is given by the formula

$$L(\bar{t}, \tilde{t}; \Delta) = p(\bar{t}, \tilde{t}; \Delta) = \prod_{g \in \{0,1\}^3} \Big(\prod_{i\colon \bar{g}_i = g} \Delta_g G(\bar{t}_i)^{\Delta_g - 1} f(\bar{t}_i) \prod_{j\colon \tilde{g}_j = g} G(\tilde{t}_j)^{\Delta_g} \Big).$$

Its logarithm is equal to

$$\begin{aligned} &\mathcal{L}(\Delta; \bar{t}, \tilde{t}) = \\ &= \sum_{g \in \{0,1\}^3} \left[S_g \log(\Delta_g) + (\Delta_g - 1) \sum_{i\colon g_i = g} \log G(\bar{t}_i) + \Delta_g \sum_{j\colon \tilde{g}_j = g} \log G(\tilde{t}_j) \right] + \sum_{i=1}^{n} \log f(\bar{t}_i) \\ &= \sum_{g \in \{0,1\}^3} \left[S_g \log \Delta_g + H_g \Delta_g \right] + \sum_{i=1}^{n} \left(\log f(\bar{t}_i) - \log G(\bar{t}_i) \right), \end{aligned}$$

where $S_g = \#\{i\colon g_i = g\}$, and $H_g = \sum_{i\colon g_i = g} \log G(\bar{t}_i) + \sum_{j\colon \tilde{g}_j = g} \log G(\tilde{t}_j)$. Clearly, the function $\mathcal{L}(\Delta; \bar{t}, \tilde{t})$ is differentiable in the whole parameter space $(0, \infty)^8$ and for each $g \in \{0,1\}^3$ the limits

$$\lim_{\Delta_g \to 0} \mathcal{L}(\Delta; \bar{t}, \tilde{t}) \quad \text{and} \quad \lim_{\Delta_g \to \infty} \mathcal{L}(\Delta; \bar{t}, \tilde{t})$$

are both equal to $-\infty$. Therefore $\mathcal{L}(\Delta; \bar{t}, \tilde{t})$ is maximized in a point where the partial derivatives $\frac{\partial}{\partial \Delta_g} \mathcal{L}(\Delta; \bar{t}, \tilde{t})$ vanish. Fortunately

$$\frac{\partial}{\partial \Delta_g} \mathcal{L}(\Delta; \bar{t}, \tilde{t}) = \frac{S_g}{\Delta_g} + H_g$$

has unique zero at $\Delta_g^{ML} = -\frac{S_g}{H_g}$, which gives the formulae for the maximum likelihood estimators.

Fix the genotypes $g_0 < g_1 < g_2 < g_3$ and consider the set

$$\mathcal{C} = \{\Delta \in \mathbb{R}^8 \colon \Delta_{g_0} \Delta_{g_3} = \Delta_{g_1} \Delta_{g_2}\}.$$

For the clarity of notation, as in the main text, we will denote the parameters of the null model in the set $\mathcal{C}$ as Δ^C. Recall that the likelihood ratio test statistic is of the form

$$\begin{aligned}\lambda(\Delta;\bar{t},\tilde{t}) &= \frac{\sup_{\Delta^C\in\mathcal{C}} L(\Delta^C;\bar{t},\tilde{t})}{\sup_{\Delta\in(0,\infty)^8} L(\Delta;\bar{t},\tilde{t})} \\ &= \frac{\sup_{\Delta^C\in\mathcal{C}}\prod_{g\in\{0,1\}^3}\Big(\prod_{i:\,\bar{g}_i=g}\Delta_g^C G(\bar{t}_i)^{\Delta_g^C-1}f(\bar{t}_i)\prod_{j:\,\tilde{g}_j=g}G(\tilde{t}_j)^{\Delta_g^C}\Big)}{\sup_{\Delta\in(0,\infty)^8}\prod_{g\in\{0,1\}^3}\Big(\prod_{i:\,\bar{g}_i=g}\Delta_g G(\bar{t}_i)^{\Delta_g-1}f(\bar{t}_i)\prod_{j:\,\tilde{g}_j=g}G(\tilde{t}_j)^{\Delta_g}\Big)}. \end{aligned} \tag{1}$$

Clearly, the denominator is maximized for $\Delta_g = \Delta_g^{ML}$. For the nominator, notice that for fixed $\Delta_{g_k}^C$, $k = 0,1,2,3$ the likelihood $L(\Delta^C,\bar{t},\tilde{t})$ is maximal if the remaining Δ_g^C's are the maximum likelihood estimators. It is therefore enough to maximize

$$\prod_{k=0}^{3}\Big(\prod_{i:\,\bar{g}_i=g_k}\Delta_{g_k}^C G(\bar{t}_i)^{\Delta_{g_k}^C-1}f(\bar{t}_i)\prod_{j:\,\tilde{g}_j=g_k}G(\tilde{t}_j)^{\Delta_{g_k}^C}\Big)$$

over $\Delta_{g_k}^C$'s satisfying $\Delta_{g_0}^C\Delta_{g_3}^C = \Delta_{g_1}^C\Delta_{g_2}^C$. Equivalently we can maximize its logarithm, and of course w can omit constants in our optimization problem, which becomes

$$\operatorname{argmax}\sum_{k=0}^{3} S_{g_k}\log\Delta_{g_k}^C + H_{g_k}\Delta_{g_k}^C. \tag{2}$$

Let us replace sub-indices g_k by k, as in the main text. Substituting Δ_3^C by $\frac{\Delta_1^C\Delta_2^C}{\Delta_0^C}$ in (2) leads to

$$\sum_{i=0}^{2}(S_i\log\Delta_i^C + H_i\Delta_i^C) + S_3(\log\Delta_1^C + \log\Delta_2^C - \log\Delta_0^C) + H_3\frac{\Delta_1^C\Delta_2^C}{\Delta_0^C}.$$

Let $\tilde{S}_1 = S_1 + S_3$, $\tilde{S}_2 = S_2 + S_3$ and $\tilde{S}_0 = S_0 - S_3$. Our goal is to maximize the function

$$D(\Delta_0^C,\Delta_1^C,\Delta_2^C)\colon = \sum_{i=0}^{2}(\tilde{S}_i\log\Delta_i^C + H_i\Delta_i^C) + H_3\frac{\Delta_1^C\Delta_2^C}{\Delta_0^C}$$

in $\Omega\colon = (0,\infty)^3$. Notice that $\lim_{\Delta\to\partial\Omega} D(\Delta^C) = -\infty$ and that D is differentiable on Ω. Thus it is enough to find zeros of its partial derivatives. Differentiating with respect to Δ_1^C we obtain

$$\frac{\partial}{\partial\Delta_1^C}D(\Delta^C) = \frac{\tilde{S}_1}{\Delta_1^C} + H_1 + H_3\frac{\Delta_2^C}{\Delta_0^C}.$$

Comparing it to 0 we infer that

$$\tilde{S}_1 + H_1\Delta_1^C = -H_3\frac{\Delta_1^C\Delta_2^C}{\Delta_0^C}. \tag{3}$$

Similarly

$$\tilde{S}_2 + H_2\Delta_2^C = -H_3\frac{\Delta_1^C\Delta_2^C}{\Delta_0^C}. \tag{4}$$

Equations (3) and (4) have the same right hand sides, hence

$$\Delta_2^C = \frac{1}{H_2}(\tilde{S}_1 - \tilde{S}_2 + H_1\Delta_1^C). \tag{5}$$

Applying the equality (5) into (3) leads to

$$\Delta_0^C = -\frac{H_3}{H_2} \cdot \frac{\tilde{S}_1 - \tilde{S}_2 + H_1\Delta_1^C}{\tilde{S}_1 + H_1\Delta_1^C}\Delta_1^C. \tag{6}$$

On the other hand, differentiating with respect to Δ_0^C gives

$$0 = \frac{\partial}{\partial \Delta_0^C} D\left(\Delta^C\right) = \frac{\tilde{S}_0}{\Delta_0^C} + H_0 - H_3\frac{\Delta_1^C\Delta_2^C}{(\Delta_0^C)^2}.$$

Multiplying by Δ_0^C and substituting Δ_0^C and Δ_2^C by the expressions from equalities (6) and (5) we obtain

$$\begin{aligned} 0 &= \tilde{S}_0 + H_0 \cdot \left(-\frac{H_3}{H_2}\right) \cdot \frac{\tilde{S}_1 - \tilde{S}_2 + H_1\Delta_1^C}{\tilde{S}_1 + H_1\Delta_1^C}\Delta_1^C \\ &\quad -H_3\Delta_1^C \cdot \frac{1}{H_2}\left(\tilde{S}_1 - \tilde{S}_2 + H_1\Delta_1^C\right) \cdot \left(-\frac{H_2}{H_3}\right) \cdot \frac{\tilde{S}_1 + H_1\Delta_1^C}{\tilde{S}_1 - \tilde{S}_2 + H_1\Delta_1^C} \cdot \frac{1}{\Delta_1} \\ &= \tilde{S}_0 - \frac{H_0H_3}{H_2} \cdot \frac{\tilde{S}_1 - \tilde{S}_2 + H_1\Delta_1^C}{\tilde{S}_1 + H_1\Delta_1^C}\Delta_1^C + \left(\tilde{S}_1 + H_1\Delta_1^C\right). \end{aligned}$$

Multiplying by the denominators gives the following equivalent form

$$\tilde{S}_0H_2(\tilde{S}_1 + H_1\Delta_1^C) - H_0H_3(\tilde{S}_1 - \tilde{S}_2 + H_1\Delta_1^C)\Delta_1^C + H_2(\tilde{S}_1 + H_1\Delta_1^C)^2 = 0.$$

And after regrouping:

$$H_1(H_1H_2 - H_0H_3)(\Delta_1^C)^2 + (H_1H_2(2\tilde{S}_1 + \tilde{S}_0) + H_0H_3(\tilde{S}_2 - \tilde{S}_1)\Delta_1^C) + H_2\tilde{S}_1^2 + H_2\tilde{S}_0\tilde{S}_1 = 0.$$

This equation, combined with equations (5), (6), and the condition $\Delta_1^C\Delta_2^C = \Delta_0^C\Delta_3^C$, may have two solutions, $(\Delta_0^C, \Delta_1^C, \Delta_2^C, \Delta_3^C)$ and $(\tilde{\Delta}_0^C, \tilde{\Delta}_1^C, \tilde{\Delta}_2^C, \tilde{\Delta}_3^C)$, and we chose the one which lays in $(0, \infty)^4$. If both do, we choose the one for which the value of the expression

$$\sum_{k=0}^{3}[S_k \log \Delta_k^C + H_k\Delta_k^C] = \log\left[\prod_{k=0}^{3}\left((\Delta_k^C)^{S_k}\prod_{i\colon \bar{g}_i=g_k} G(\bar{t}_i)^{\Delta_k^C}\prod_{j\colon \tilde{g}_j=g_k} G(\tilde{t}_j)^{\Delta_k^C}\right)\right]$$

is greater.

Finally, inserting the estimated maximizing parameters into the nominator of the likelihood ratio (1), we obtain

$$\prod_{k=0}^{3}\left(\prod_{i\colon \bar{g}_i=g_k}\Delta_g^C G(\bar{t}_i)^{\Delta_g^C-1}f(\bar{t}_i)\prod_{j\colon \tilde{g}_j=g_k} G(\tilde{t}_j)^{\Delta_g^C}\right)\prod_{g\notin\{g_0,g_1,g_2,g_3\}}\left(\prod_{i\colon \bar{g}_i=g}\Delta_g G(\bar{t}_i)^{\Delta_g-1}f(\bar{t}_i)\prod_{j\colon \tilde{g}_j=g} G(\tilde{t}_j)^{\Delta_g}\right),$$

while inserting the estimated maximizing parameters into the denominator gives

$$\prod_{k=0}^{3}\left(\prod_{i\colon \bar{g}_i=g_k}\Delta_g G(\bar{t}_i)^{\Delta_g-1}f(\bar{t}_i)\prod_{j\colon \tilde{g}_j=g_k} G(\tilde{t}_j)^{\Delta_g}\right)\prod_{g\notin\{g_0,g_1,g_2,g_3\}}\left(\prod_{i\colon \bar{g}_i=g}\Delta_g G(\bar{t}_i)^{\Delta_g-1}f(\bar{t}_i)\prod_{j\colon \tilde{g}_j=g} G(\tilde{t}_j)^{\Delta_g}\right).$$

Thus, in the ratio (1) the expressions for $g \notin \{g_0, g_1, g_2, g_3\}$, as well as the terms $\prod_{k=0}^{3}\prod_{i\colon g_i=g_k} f(\bar{t}_i)$ occur both in the nominator and in the denominator and cancel out. Multiplying both the nominator and the denominator by $\prod_{k=0}^{3}\prod_{i\colon g_i=g_k} G(\bar{t}_i)$, we obtain the likelihood ratio of the form

presented in the main text

$$\lambda(\Delta,\bar{t},\tilde{t}) = \frac{\prod_{k=0}^{3}\left(\prod_{i:\ \bar{g}_i=g_k}\Delta_k^C G(\bar{t}_i)^{\Delta_k^C}\prod_{j:\ \tilde{g}_j=g_k}G(\tilde{t}_j)^{\Delta_k^C}\right)}{\prod_{k=0}^{3}\left(\prod_{i:\ \bar{g}_i=g_k}\Delta_k G(\bar{t}_i)^{\Delta_k}\prod_{j:\ \tilde{g}_j=g_k}G(\tilde{t}_j)^{\Delta_k}\right)}.$$
